# Cross-Sectional Comparative Analysis of Gut Microbiota in Spanish Adolescents with Mediterranean and Western Diets

**DOI:** 10.3390/nu17030388

**Published:** 2025-01-22

**Authors:** Marina Redruello-Requejo, María del Mar Blaya, Daniel González-Reguero, Marina Robas-Mora, Javier Arranz-Herrero, Teresa Partearroyo, Gregorio Varela-Moreiras, Diana Penalba-Iglesias, Pedro Jiménez-Gómez, Paloma Reche-Sainz

**Affiliations:** 1 Grupo USP-CEU de Excelencia “Nutrición para la vida (Nutrition for Life)”, Ref: E02/0720, Department of Pharmaceutical and Health Sciences, Faculty of Pharmacy, San Pablo University, CEU Universities, Urbanización Montepríncipe, 28660 Boadilla del Monte, Spain; t.partearroyo@ceu.es (T.P.); gvarela@ceu.es (G.V.-M.); 2 Instituto Universitario CEU Alimentación y Sociedad, Faculty of Pharmacy, San Pablo University, CEU Universities, Urbanización Montepríncipe, 28660 Boadilla del Monte, Spain; 3 Department of Pharmaceutical and Health Sciences, San Pablo University, CEU Universities, Urbanización Montepríncipe, 28660 Boadilla del Monte, Spain; mar.blayaperalta@usp.ceu.es (M.d.M.B.); daniel.gonzalezreguero@ceu.es (D.G.-R.); marina.robasmora@ceu.es (M.R.-M.); j.arranz3@usp.ceu.es (J.A.-H.); diana.penalbaiglesias@ceu.es (D.P.-I.); paloma.rechesainz@ceu.es (P.R.-S.); 4 Departamento de Ciencias Médicas Básicas, Instituto de Medicina Molecular Aplicada (IMMA) Nemesio Díez, Medicine Faculty, San Pablo University, CEU Universities, Urbanización Montepríncipe, 28660 Boadilla del Monte, Spain

**Keywords:** microbiota, microbiome, microbial diversity, Shannon index, Mediterranean diet, Western diet, ultra-processed foods, 16S rRNA, antibiotic resistance, cenoantibiogram

## Abstract

Dietary patterns, such as the Mediterranean diet (MD) and the Western diet (WD), influence gut microbiota composition and functionality, which play important roles in energy metabolism and nutrient absorption. Objectives: A descriptive cross-sectional study was designed to evaluate the gut microbiota of 19 Spanish adolescents and to investigate the association of MD and ultra-processed food (UPF) intake with microbial diversity and community structure. Methods: Functional diversity of gut microbiota was evaluated using Biolog EcoPlates, taxonomic composition was assessed with 16S rRNA sequencing via MinION, and phenotypic responses to antibiotics were analyzed using the cenoantibiogram technique under aerobic and anaerobic conditions. Results: Adolescents with higher adherence to the MD exhibited greater functional diversity, as per the Shannon–Weaver index. In addition, this group showed higher abundance of bacterial genera previously described as beneficial, such as *Paraclostridium*, *Anaerobutyricum*, *Romboutsia*, and *Butyricicoccus*. In contrast, adolescents reporting greater UPF intakes had a microbiota composition similar to those with low adherence to the MD, characterized by decreased abundance of beneficial genera. Regarding antibiotic resistance, significant differences were only observed under anaerobic conditions, with individuals with low adherence to the MD showing more sensitivity for most antibiotics tested. Conclusions: These results suggest that the MD promotes a healthier and more balanced gut environment, potentially improving metabolic functions in adolescents. Despite the lack of differences in α-diversity, comparisons of microbial community structure between adolescents following the MD and those with high UPF (characteristic of the WD) showed clear differences in terms of β-diversity. These findings suggest that dietary patterns influence the composition of the gut microbiota in a more complex manner, beyond just taxonomic richness. The outcomes of this exploratory study highlight opportunities for future research to deepen understanding of the long-term health implications of these dietary patterns, as well as the mechanisms regulating the composition, functionality, and phenotypic responses to antibiotics of gut microbial communities.

## 1. Introduction

The central role of the gut microbiota as an integrative axis of human health and homeostasis is widely recognized today. The human microbiota is a highly diverse ecosystem, composed primarily of bacteria (90%), but also viruses, fungi, archaea, and protozoa [1]. Through its genetic load and metabolic products, this ecosystem plays a key role in regulating functions such as vitamin and short-chain fatty acid (SCFA) synthesis, lipid oxidation, fat storage, prevention of colonization by enteropathogenic bacteria, and maturation and regulation of the immune system and the gut–brain axis [2,3,4,5].

The gut microbiota is largely determined by maternal transmission, although it undergoes changes throughout life influenced by a variety of factors [6]. Among these factors, diet plays a crucial role in determining the composition and functionality of microbial communities. Dietary patterns can promote a state of eubiosis, characterized by the balance of beneficial bacteria, or conversely, disrupt this balance, leading to a state of dysbiosis. This microbial imbalance is associated with the development of various metabolic, gastrointestinal, and even neurodegenerative diseases [7].

In this context, the Western diet (WD), rich in ultra-processed foods (UPFs), has been linked to alterations in the gut microbiota, favoring an inflammatory profile and a higher incidence of various chronic non-communicable diseases [7,8]. Indeed, in the prospective SUN cohort study conducted in Spain, it was observed that higher UPF consumption was independently associated with a 62% relative increase in the risk of all-cause mortality [9].

Specifically, UPFs refer to food products that typically contain five or more ingredients, which primarily represent cheap industrial sources of energy with low nutritional density. Their most common ingredients include refined sugars and fats, along with other food additives such as emulsifiers, sweeteners, or colorants. Furthermore, UPFs are often characterized by low fiber content and a lack of essential nutrients [10]. This contrasts with the Mediterranean Diet (MD), which emphasizes fresh, minimally processed foods and is characterized by a high content of dietary fiber, polyphenols, and unsaturated fatty acids. This dietary pattern promotes a healthy microbial profile and reduces the risk of metabolic and cardiovascular diseases [11].

Alterations in the gut microbiota have also been associated with increased antibiotic resistance [4], a growing public health issue that could be exacerbated by dietary patterns unfavorable to maintaining a healthy intestinal environment. The World Health Organization (WHO) estimates that, by 2050, deaths related to antibiotic resistance could reach 10 million annually, surpassing deaths caused by cancer [12]. The relationship between antibiotics and the gut microbiome is intricate and multifactorial, playing a crucial role in the development of antibiotic resistance. Tackling this challenge demands a thorough understanding of microbial dynamics and the creation of new strategies to protect microbiome health and curb the transmission of resistant genes.

Understanding how Western dietary patterns and UPF intake affect the gut microbiota, and its metabolic functions could be key to developing dietary and probiotic interventions to mitigate these negative effects, contributing to both the prevention and the treatment of various infectious and/or chronic diseases.

This study aims to analyze the impact of the MD compared to the WD on the gut microbiota of adolescents, exploring its relationship with microbial functional diversity, antibiotic response, and the taxonomic composition of microbial communities. This analysis could provide insights into the influence of dietary patterns on gut health and their implications for long-term metabolic health.

## 2. Materials and Methods

### 2.1. Study Design and Sample Selection

A descriptive cross-sectional study was conducted in a cohort of *n* = 19 adolescents, aged between 13 and 17 years, all residents of the Autonomous Community of Madrid.

A stratified random sampling was applied based on the variables age and sex. The selection of participants was carried out randomly at the street level, according to the following criteria:

– Inclusion criteria: healthy individuals aged 13 to <18 years, following their usual diet, whose parents/guardians were able to read and understand the questionnaires and agreed to sign the informed consent.
– Exclusion criteria: individuals that did not follow their usual diet at the time of the fieldwork, that received antibiotic or probiotic treatment in the previous month, that did not provide a signed consent form for participation, or that did not complete any phase of the study.

The study protocol was approved by the Research Ethics Committee of Universidad San Pablo-CEU with code 578/22/55. Informed consent was obtained from all participants prior to their involvement in the study, and recommendations included in the Declaration of Helsinki were always followed [13]. The fieldwork was conducted between January and February 2023 at the facilities of the Universidad San Pablo-CEU (Madrid). Final sample size implies a ±20% margin of error for a 95.5% confidence interval, with the estimation of equally probable categories (p = q = 50%), considering a population of 6 million children and adolescents in Spain according to the 2020 census data published by the National Statistics Institute (INE) [14].

This research adheres to the STROBE Guidelines [15] (Supplementary Materials).

### 2.2. Diet Quality Assessment

To evaluate the overall quality of the diet, participants completed two dietary questionnaires that assess adherence to the Mediterranean diet (MD) (KIDMED Score) [16] and the consumption of ultra-processed food (UPF) (SQ-HPF Score) [17].

For each dietary score, participants were divided into two groups based on the median score of all participants (as no significant differences were found for any of the scores according to sex): Adherence to MD was divided into “LowMD” and “HighMD”, while UPF intake was divided into “LowUPF” and “HighUPF”.

### 2.3. Analysis of Gut Microbiota

A cecal sample was collected from each participant. Samples were immediately refrigerated on ice and stored at −80 °C until further analysis.

#### 2.3.1. Comparative Functional Analysis

For each participant, 2 g of cecal sample was suspended in a 0.45% sterile saline solution to a final volume of 20 mL. The density of viable microorganisms was confirmed to be cfu/mL (optical density [OD] = 0.5 McFarland). From the obtained microbial suspension, 135 µL per well were loaded into Biolog Ecoplate™ plates (Biolog Inc., Hayward, CA, USA), generating a technical replicate (*n* = 3). The plates were incubated at 37 °C and analyzed every 24 h. Absorbance was measured at 630 nm and 595 nm using the Asys UVM340 plate reader and Micro Win™ V3.5 software. The comparative functional analysis was performed using the corrected absorbance values from the chosen incubation time as average well color development (AWDC) [18], and the metabolic diversity of each sample was calculated using the Shannon–Weaver diversity index. All procedures were conducted as described in the protocol [19].

#### 2.3.2. Comparative Analysis of Antibiotic Response

For each sample, the same bacterial suspension obtained under the conditions described in the previous section was plated on Mueller-Hinton agar (Condalab^®^, Madrid, Spain). Antibiotic resistance was evaluated with the Epsilon test method (ε-test) as the Minimum Inhibitory Concentration (MIC) from cultures under both anaerobic and aerobic conditions, according to the model described by Marina Robas Mora et al. [20] Plates were incubated following the manufacturer’s instructions, and the most restrictive inhibition zones were measured after 48 h of incubation.

In aerobic cultures, the following antibiotics were tested: amikacin (AK), amoxicillin (AML), cefpirome (CR), ceftazidime (CAZ), gentamicin (CN), and sulfamethoxazole/trimethoprim (TS) (BioMérieux^®^, Marcy l’Etoile, France). Anaerobic cultures were incubated in an anaerobic bag system (Merck KGaA, 64271, Darmstadt, Germany), and the tested antibiotics included the following: amoxicillin/clavulanic acid (AUG), azithromycin (AZM), cefoxitin (FOX), ciprofloxacin (CIP), clindamycin (CD), imipenem (IMI), IMI+EDTA, metronidazole (MTZ), rifampicin (RD), and levofloxacin (LEV) (BioMérieux^®^, Marcy l’Etoile, France).

Due to a limitation in the amount of sample provided, *n* = 3 cenoantibiogram tests could not be performed under anaerobic conditions.

#### 2.3.3. Metagenomic Analysis

Microbial DNA was extracted using the Real Microbiome Fecal DNA Kit (Real Laboratory SL, Valencia, Spain). DNA concentration was measured using a Nanodrop2000 and adjusted to 10 μg with nuclease-free water. DNA was amplified using the 16S Barcoding Kit (SQK-RAB204, Oxford Nanopore Technology, Oxford, UK). The primers used for the amplification of the 16S rRNA region are specific for the full region, using primers 27F and 1492R. The PCR product was purified using the “HighPrep™ PCR Bead Purification” (MagBio Genomics, Gaithersburg, MD, USA). Next, the library was prepared according to the instructions of the 16S Barcoding Kit. A total of 200 μg of samples were loaded onto an R9.4.1 flow cell and sequenced on the MinION Mk1C device for 12 h.

### 2.4. Statistical Analysis

Results from the characterization of the sample are presented as absolute (n) and relative (%) frequencies. The Shapiro–Wilk normality test was applied to assess the distribution of the studied variables, and comparisons between groups were determined by the Student’s *t*-test or the Mann–Whitney U-test, accordingly. Significance was set at *p* < 0.05, with a 95% confidence level.

For the comparative analysis of the antibiotic response, the two variables that best explain the model were projected onto the two-dimensional plane to study potential groupings between treatments, based on principal component analysis (PCA).

Finally, the results of the metagenomic analysis were processed using the Fastq 16S v2022.01.07 workflow from EPI2ME (Oxford Nanopore Technologies, Oxford, UK) with default settings. The results were analyzed using RStudio, generating the relative abundances. A- and β-diversity analyses were obtained using the MicrobiomeStat package, as well as the differential abundance analysis. α-diversity was calculated using the Chao1, Shannon, and Simpson indices, allowing visualization of diversity within a sample. β-diversity was calculated using a principal coordinates analysis (PCoA) on distances calculated with Bray–Curtis (BC) (BCij = 1 − (2 × Cij)/(Si + Sj)) and Jaccard (J (A, B) = | A∩B |/| A∪B |) between samples. PCoA allows visualization of differences between groups in a dimensional space.

Statistical analyses were performed with IBM SPSS v29.0 Statistics (IBM Corp., Armonk, NY, USA).

## 3. Results

A general description of the characteristics of the studied population is provided in Table 1.

All comparative analyses were conducted based on the dietary patterns of adherence to the MD (classified as HighMD and LowMD) and WD (classified as HighUPF and LowMD).

### 3.1. Comparative Functional Analysis

The results from the Biolog Ecoplate™ assay showed that the peak of metabolic activity was reached at 144 h. The comparative functional analysis was conducted considering the metabolic activity at this time point.

**Figure 1:**
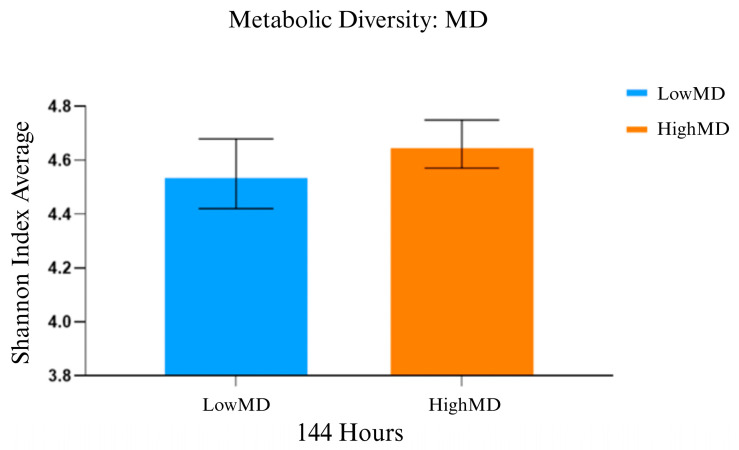
Metabolic diversity of gut microbiota according to the degree of adherence to the Mediterranean diet. Bar chart for the two MD adherence groups represented on the X-axis, measured after 144 h. The Y-axis shows the mean values of the Shannon–Weaver index, with error bars indicating variability within each group. MD: Mediterranean diet.

Metabolic diversity was assessed using the Shannon–Weaver diversity index, for which the following means and standard deviations were obtained:

The HighMD group (Figure 1) showed 4.66 ± 0.089, compared to the LowMD group, which showed 4.55 ± 0.13. No statistically significant differences were identified ($t_{17}$ = −0.739; *p* = 0.470), although a higher metabolic diversity index was observed in HighMD compared to LowMD at specific points.

On the other hand, the results for HighUPF and LowUPF (Figure S1) did not show statistically significant differences ($t_{17}$ = −0.717 *p*= 0.483).

### 3.2. Comparative Analysis of Antibiotic Response

The data analysis of the antibiotic resistance profile using the cenoantibiogram provided the MICs for each sample under aerobic and anaerobic conditions (Tables S1 and S2). PCA was performed to observe trends and behaviors of the microbiota at the population level, based on the studied dietary patterns.

#### 3.2.1. Aerobic Conditions

**Figure 2:**
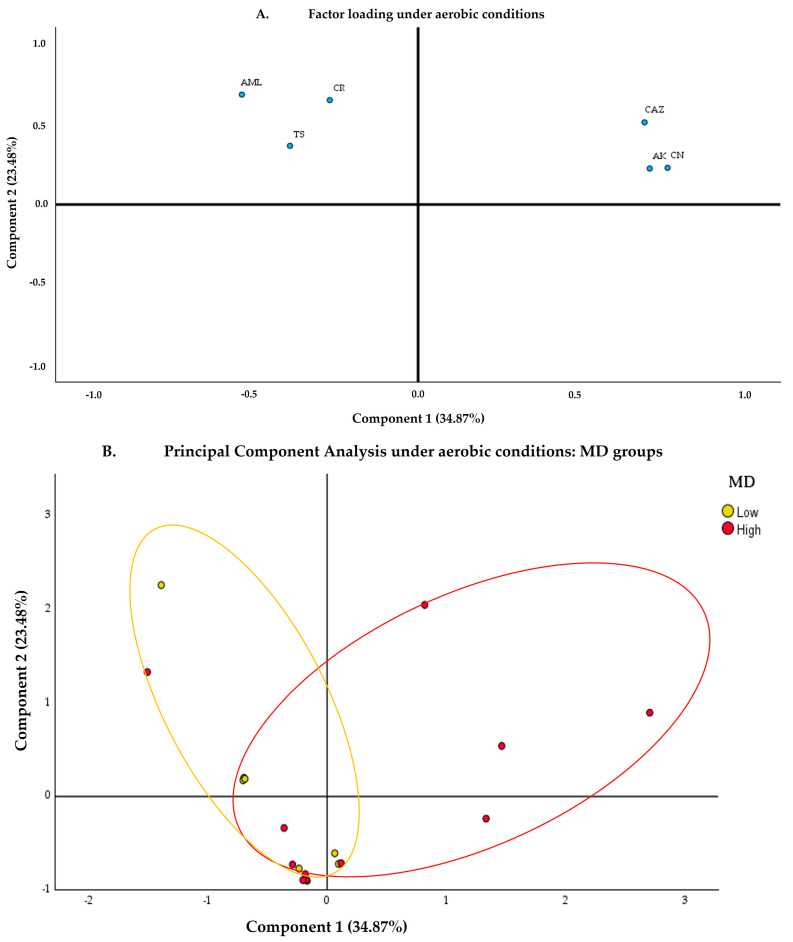
Antibiotic response under aerobic conditions according to the degree of adherence to the Mediterranean diet. (**A**) Factor loading plot under aerobic conditions. The X-axis (component 1) represents the first principal component, which explains 34.87% of the variance, while the Y-axis (component 2) represents the second principal component, explaining 23.48% of the variance. The data points are labeled (AK, AML, CAZ, CN, CR, and TS), indicating the different antibiotics contributing to these components. (**B**) The PCA under aerobic conditions shows the points based on the LowMD and HighMD groups, as indicated in the legend. The ellipses represent confidence intervals containing approximately 95% of the observations for each group, thus showing the clustering of data points within each group. AK: amikacin; AML: amoxicillin; CAZ: ceftazidime; CN: gentamicin; CR: cefpirome; MD: Mediterranean diet; PCA: principal component analysis; TS: sulfamethoxazole/trimethoprim.

Factor loadings (Figure 2A) allow for the interpretation of how antibiotics contribute to the principal components and how these components have a greater or lesser impact on the gut microbiota, depending on the groups being studied. The antibiotics CAZ, AK, and CN show a significant contribution to component 1. At the same time, AML, CR, and TS have a high positive load on component 2, indicating that they also contribute significantly.

The PCA ellipses (Figure 2B) show an area of overlap, although the LowMD and HighMD groups are differentiated. The HighMD group tends to cluster towards the right of component 1 and towards the upper part of component 2, while the LowMD group is more dispersed towards the left of component 1 and the lower part of component 2. The dispersion of points within each group indicates internal variability. HighMD shows a higher concentration of points, while LowMD is more spread out.

The variances explained by component 1 showed low MIC values (Table S1) for the antibiotics CAZ, AK, and CN in the HighMD and LowMD groups. On the other hand, the variances explained by component 2, for the antibiotics AML, CR, and TS, generally showed lower MICs, although some heterogeneity was observed for both HighMD and LowMD. Additionally, samples from both groups were observed to cluster near the axis (0,0), suggesting that their variability does not present extreme characteristics compared to other samples.

When comparing the LowMD and HighUPF groups (Figure S2), representing the WD, very similar trends were observed between the groups. Both LowMD and HighUPF are positioned in the left area, showing trends along component 2.

#### 3.2.2. Anaerobic Conditions

**Figure 3:**
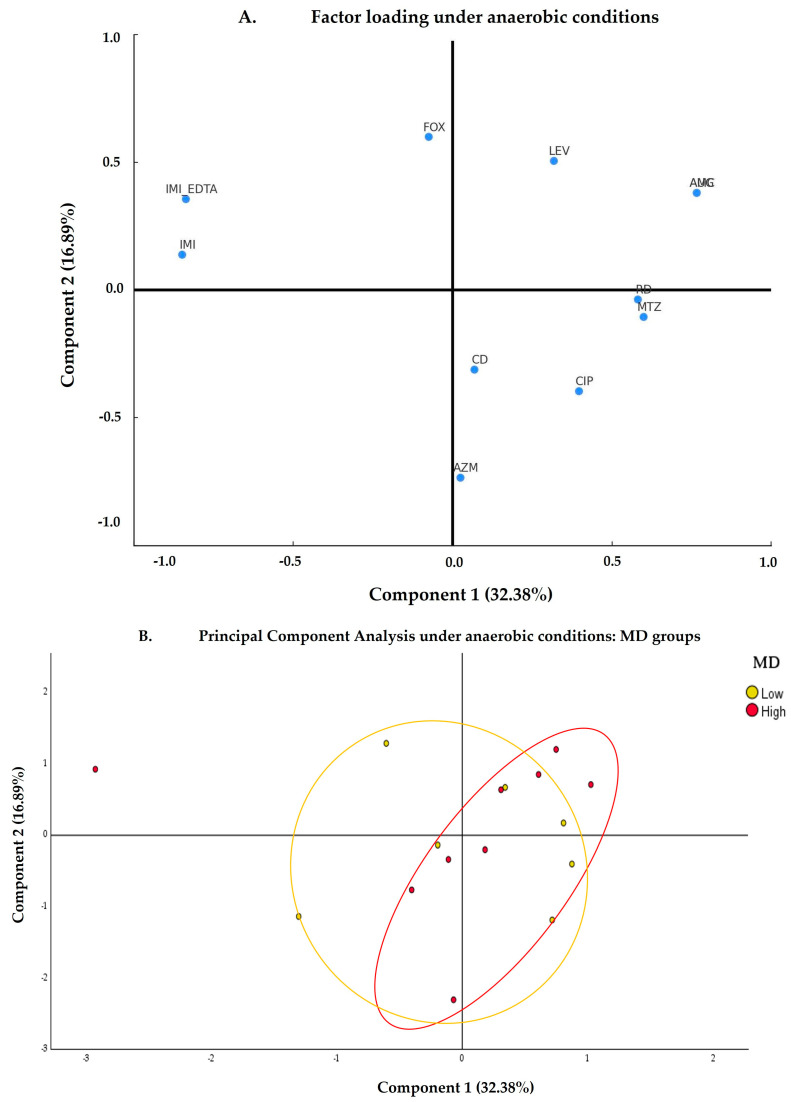
Antibiotic response under anaerobic conditions according to the degree of adherence to the Mediterranean Diet. (**A**) Factor loading plot under anaerobic conditions. The X-axis (component 1) represents the first principal component, which explains 32.38% of the variance, while the Y-axis (component 2) represents the second principal component, explaining 15.37% of the variance. The data points are labeled (AUG, AZM, CD, CIP, FOX, IMI, IMI+EDTA, LEV, MTZ, and RD), indicating the different antibiotics contributing to these components. (**B**) The PCA under anaerobic conditions shows the points differentiated between the LowMD and HighMD groups, as indicated in the legend. The ellipses represent the confidence intervals containing approximately 95% of the observations for each group. AUG: amoxicillin/clavulanic acid; AZM: azithromycin, CD: clindamycin; CIP: ciprofloxacin; FOX: cefoxitin; IMI: imipenem; IMI+EDTA: imipenem+EDTA; LEV: levofloxacin; MD: Mediterranean diet; MTZ: metronidazole; PCA: principal component analysis; RD: rifampicin.

For bacteria cultured under anaerobic conditions, the antibiotics IMI, IMI EDTA, and FOX are grouped in the upper left quadrant (Figure 3A), indicating that they have a significant load on component 2. On the other hand, LEV and AMG have a high load on both components, suggesting that these antibiotics are important for explaining the variability observed in the gut microbiota in principal components 1 and 2. Finally, AZM, CIP, CD, MTZ, and RD have a high load on component 1. The distance of these antibiotics from the origin suggests that they have a strong influence on the structure of the microbiota.

The PCA ellipses (Figure 3B) show a certain trend of separation between the LowMD and HighMD groups. However, there is some overlap between them. The HighMD group tends to cluster towards the right of component 1, showing a concentration of points, although they are also spread out in the lower part of the quadrants. On the other hand, the LowMD group is dispersed widely from the coordinate axis (0,0) across the four quadrants, showing a more heterogeneous distribution.

The variances explained by component 1 showed higher MICs (Table S2) in the LowMD group for the antibiotics AZM, CIP, CD, MTZ, and RD, while for LEV and AUG, the values were lower. In the HighMD group, for most of the antibiotics studied (LEV, AUG, AZM, CIP, CD, MTZ, and RD), reduced MICs were observed for most individuals in this group. Additionally, the variances explained by component 2 presented higher MICs for the HighMD group compared to the LowMD group for the antibiotics IMI and IMI+EDTA.

The WD, represented by the HighUPF (Figure S3) and LowMD groups, showed very similar patterns. Both are represented in the left area, showing trends in component 2.

### 3.3. Metagenomic Analysis

A metagenomic analysis was performed using 16S rRNA amplicon sequencing of the full region. High-throughput sequencing of DNA extracted from the 19 cecal samples generated a total of 1,864,096 high-quality reads. In the LowMD group, 777,677 reads were obtained with an average of 86,408.5 per sample. In the HighMD group, 875,391 reads were obtained with an average of 87,539.1. Meanwhile, 846,640 reads were obtained in the LowUPF group with an average of 120,946.286. In the HighUPF group, 806,444 reads were generated with an average of 67,203.6.

#### 3.3.1. Relative Abundances

**Figure 4:**
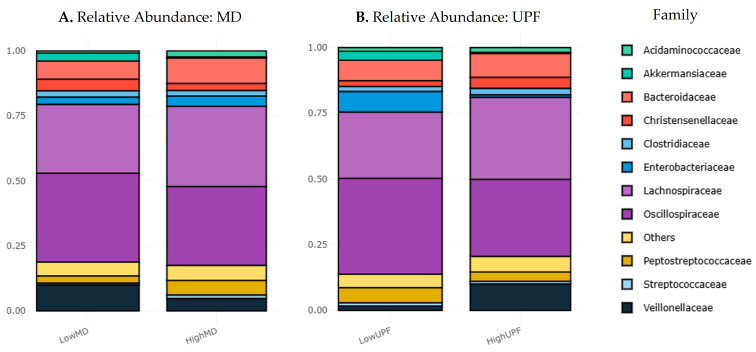
Relative abundance in the gut microbiota at the family level. The Y-axis represents the proportion within each microbial community, with values ranging from 0.00 to 1.00 (fractional). The X-axis differs between two groups. The different colors represent the 11 most significant families, listed in the legend. (**A**) Shows the comparison of relative abundances between LowMD and HighMD. (**B**) Shows the comparison of relative abundances between LowUPF and HighUPF. MD: Mediterranean diet; UPF: ultra-processed foods.

The relative abundances of the intestinal microbial composition at the family level were compared (Figure 4).

The detailed relative abundances at different taxonomic levels are shown in Tables S3 and S4 for the MD groups and UPF groups, respectively. At the family level, the abundances displayed very similar profiles between the HighMD group (Figure 4A) and the LowUPF group (Figure 4B), representative of the WD, as well as between LowMD and HighMD. No statistically significant differences were observed in the MD group (U = 74, *p* = 0.931) or in the UPF group (U = 73, *p* = 0.977). However, large percentage changes between groups were noted.

In the MD group (Figure 4A), the families *Oscillospiraceae* (LowMD = 34.12% and HighMD = 30.32%), *Veillonellaceae* (LowMD = 9.98% and HighMD = 4.69%), and *Akkermansiaceae* (LowMD = 3% and HighMD = 0.38%) increased in LowMD. Meanwhile, relative abundances increased in HighMD for the families *Lachnospiraceae* (LowMD = 26.44% and HighMD = 30.87%), *Enterobacteriaceae* (LowMD = 2.8% and HighMD = 4%), and *Peptostreptococcaceae* (LowMD = 2.72% and HighMD = 5.59%).

On the other hand, in the UPF group (Figure 4B), increases in LowUPF were observed for the families *Oscillospiraceae* (LowUPF = 36.45% and HighUPF = 29.28%), *Enterobacteriaceae* (LowUPF = 7.86% and HighUPF = 0.92%), *Akkermansiaceae* (LowUPF = 3.38% and HighUPF = 0.42%), and *Peptostreptococcaceae* (LowUPF = 5.73% and HighUPF = 3.59%). In contrast, increases in HighUPF were observed for the families *Lachnospiraceae* (LowUPF = 25.18% and HighUPF = 31.24%) and *Veillonellaceae* (LowUPF = 1.65% and HighUPF = 10%).

A correlation was observed between LowMD and HighUPF, representative of the WD, in the families *Enterobacteriaceae*, *Veillonellaceae*, and *Peptostreptococcaceae*.

#### 3.3.2. α- and β-Diversity

**Figure 5:**
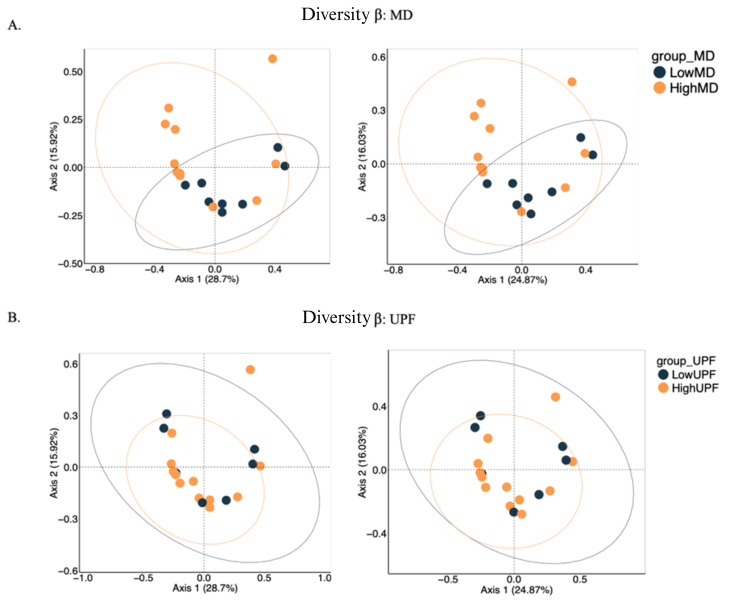
β-diversity for (**A**) MD and (**B**) UPF groups. The PCoAs on the left were derived using Bray–Curtis, while those on the right were obtained using Jaccard. The multidimensional components are reduced to two, represented on the Y-axis (Axis 2) and the X-axis (Axis 1). Both axes represent the variability of the data. Each point on the plot represents an individual sample. The position of the points reflects the microbial composition of each sample. Points of different colors represent different groups, as shown in the legends. The ellipses surrounding the points represent 95% confidence intervals for each group, indicating dispersion and similarity. MD: Mediterranean diet; PCoA: principal coordinates analysis; UPF: ultra-processed foods.

To explore alterations in the microbial community structure among adolescents and the impact of dietary patterns, α-diversity analysis (Figure S4) was performed, measuring both richness and diversity within a sample, and β-diversity (Figure 5), comparing differences in species composition between samples, all at the genus level.

The α-diversity indices in the MD adherence group (Figure S4A) did not show statistically significant differences, although a more diverse distribution was observed in the HighMD group.

To assess the impact of the WD, α-diversity indices were compared between the UPF group (Figure S4B) and the MD group (Figure S4A). No statistically significant differences or clear associations were observed between the HighUPF and LowUPF groups. However, a slight increase in diversity and evenness was recorded in the HighUPF group.

β-diversity with respect to MD adherence (Figure 5A) shows greater separation between the LowMD and HighMD points. This suggests more pronounced differences in microbiota composition between the samples. The lack of significant overlap between the 95% confidence ellipses suggests these differences are statistically significant.

When visualizing the HighUPF and LowMD groups (Figure 5B), typical of the WD, similar behaviors were observed with trends towards grouping, although the UPF group did not show as clear separation as the MD group.

The variances extracted from both methods are very similar, considering relative abundances (BC) and considering the presence/absence of bacteria (Jaccard), thus indicating a consistent representation of the results.

#### 3.3.3. Diversity at the Genus Level

**Figure 6:**
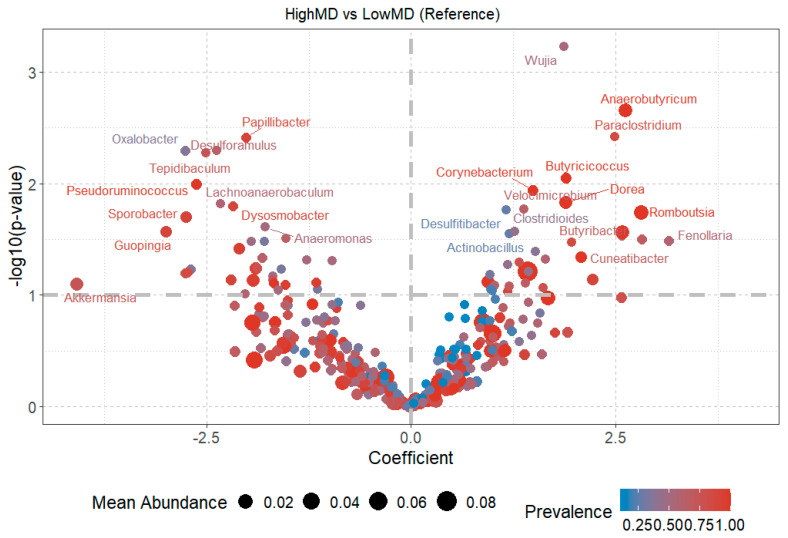
The volcano plot presents the fold change coefficient on the X-axis between the HighMD and LowMD groups. Positive values indicate higher abundance in the HighMD group, while negative values indicate higher abundance in the LowMD group. The Y-axis shows the value of −log10 (*p*-value), with values greater than 1 indicating statistically significant differences between groups. MD: Mediterranean diet.

After performing differential abundance analysis (DAA), differences in bacterial abundance at the genus level were found for the HighMD and LowMD groups (Figure 6). Particularly, higher abundance is observed in the HighMD group for the genera *Dorea*, *Anaerobutyricum*, *Romboutsia*, *Clostridioides*, *Paraclostridium*, and *Butyricicoccus*, among others. In contrast, the genera *Lachnoanaerobaculum*, *Oxalobacter*, *Dysosmobacter*, *Sporobacter*, and *Tepidibaculum*, among others, decreased compared to LowMD.

**Figure 7:**
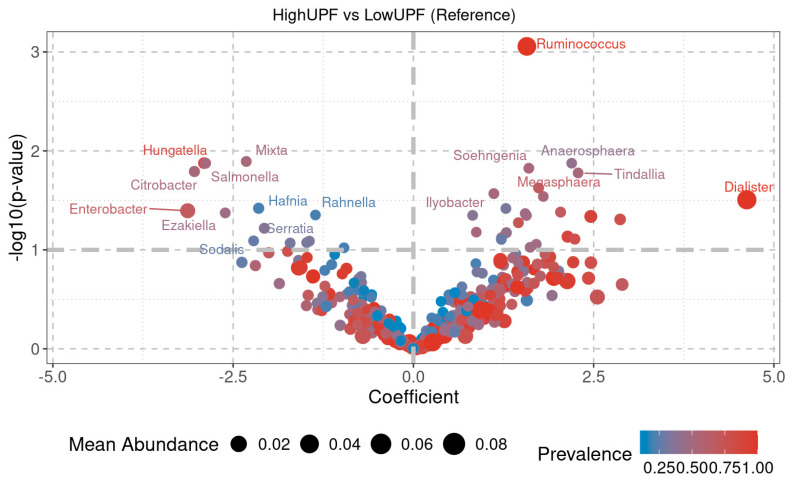
The volcano plot shows the fold change coefficient on the X-axis between the two compared groups: HighUPF and LowUPF. Positive values indicate higher abundance in the HighUPF group, while negative values indicate higher abundance in the LowUPF group. The Y-axis shows the value –log10 (*p*-value), with values greater than 1 indicating statistically significant differences between the groups. UPF: ultra-processed foods.

For the HighUPF and LowUPF groups (Figure 7), statistically significant differences are also observed between various genera. Higher mean abundance is seen in the genera *Dialester*, *Ruminococcus*, *Megasphaera*, *Anaerosphaera*, and *Tindallia*, among others, in the HighUPF group, while genera such as *Hungatella*, *Enterobacter*, *Salmonella*, *Citrobacter*, *Hafnia*, *Rahnella*, *Serratia*, *Ezakitella*, and *Sodalis*, among others, are decreased in comparison to the LowUPF group.

A greater number of genera with statistically significant differences were observed in the MD groups (Figure 6) compared to the UPF groups (Figure 7).

## 4. Discussion

The gut microbiota has evolved from being considered a largely passive community of commensal microorganisms to being studied as an active and dynamic microbial community with a significant impact on nearly all areas of human physiology. The relationship between gut microbiota and cardiometabolic diseases, such as obesity and T2D, has been widely documented [21].

It is estimated that approximately 60% of gut microbiota structure is influenced by diet, highlighting the importance of dietary habits in shaping gut microbial ecology [22]. Dietary patterns can influence both the composition and function of these microbial communities, contributing to the maintenance of eubiosis or, conversely, triggering dysbiosis. Recently, comparisons between populations with distinctly different diets have revealed differences in the characteristics of the gut microbiota. In general, populations living in urban areas or with some degree of industrialization tend to show reduced diversity in the gut microbiome [23,24,25]. As a result, a recent study revealed that 124 bacterial species are disappearing in today’s industrialized gut microbiome when compared to that of hunter-gatherers [26].

In this context, the present study evaluated the effects of the MD and WD on the gut microbiota of a group of Spanish adolescents.

The MD has been widely recognized for its preventive properties against non-communicable diseases. Characterized by low consumption of processed foods and a high intake of foods rich in macronutrients, antioxidants, and complex insoluble fiber, it was the first diet studied in this context [27]. Metagenomic studies have suggested that the high fiber intake typical of the MD promotes beneficial modulation of certain microbial taxa, contributing to host health protection [28].

On the other hand, the WD, defined by its high content of refined sugars, fats, and UPF, has been associated with intestinal permeability and systemic low-grade inflammation [29]. Specific components of Western-style diets directly modulate metabolism or immune responses, or indirectly affect inflammatory phenotypes by altering the composition or function of the gut microbiota. For example, in healthy volunteers, endotoxemia induced by a WD (i.e., an increase in serum concentrations of lipopolysaccharides derived from *Bacteroides* or *Prevotella* species) promotes inflammatory processes, while microbial rarefaction encourages calorie storage in the host. Consequently, chronic inflammation promotes metabolic and immune-mediated diseases [7].

In fact, the notion that disbalanced human gut microbiota promotes disease is supported by the transmissibility of phenotypes from patients with obesity, steatotic liver disease, inflammatory bowel disease, and colorectal cancer into mice via microbial transplantation [30,31,32,33]. Moreover, findings from the PREDICT-1 Trial [34], in which gut microbiomes of 1098 participants were analyzed, revealed that the diversity and quality of a healthy diet could be predicted based on the gut microbiome. In addition, overall microbiome composition was predictive for several cardiometabolic blood markers, including glycemic, lipemic, and inflammatory indices [34]. In this regard, probiotics offer a promising therapeutic approach for the management of a wide array of diseases and warrant consideration as a potential adjunct therapy in clinical practice. For example, results from a recent systematic review show how probiotics could offer potential benefits in terms of glycemic control, insulin sensitivity, and inflammation reduction in the management of diabetic patients [35]. For metabolic-associated fatty liver disease (MAFLD), another systematic review on randomized controlled trials showed that probiotic supplementation can reduce liver enzyme levels and regulate glycometabolism [36].

In our study, adherence to the MD demonstrated an increase in the functional diversity of gut microbiota in the HighMD group compared to the LowMD group (Figure 1). Although no greater variety of microbial species was observed, the HighMD group exhibited a more balanced distribution in terms of α-diversity (Figure S4A). Moreover, significant differences in β-diversity were identified between the two groups (Figure 5A), indicating variations in the composition of the gut microbiota. These findings are consistent with previous studies suggesting a positive association between adherence to the MD and greater gut microbiota diversity [37].

Overall, these results suggest that the increased functional diversity and beneficial microbial modulation observed in the HighMD group are closely related to nutrients characteristic of the MD. The high dietary fiber intake characteristic of the MD not only benefits microbial diversity but also promotes the production of SCFAs, such as acetate, butyrate, and propionate, through fermentation processes. These compounds play a crucial role in maintaining gut health [38]. Additionally, adherence to the MD, combined with high consumption of fiber, legumes, vegetables, fruits, and nuts, is associated with an increase in butyrate-producing taxa abundance, further reinforcing its anti-inflammatory benefits [39].

On the other hand, prospective analysis of population distributions through the cenoantibiogram technique, based on variance results explained by PCA, indicated differentiation between HighMD and LowMD groups under aerobic (Figure 2) and anaerobic conditions (Figure 3). These findings suggest that dietary patterns may impact both the taxonomic composition and phenotypic expression of the gut microbiota.

Previous studies have revealed that mice consuming a high-fat diet are less sensitive to antibiotic treatment following infection compared to those on a standard diet. Conversely, a high-fiber diet, such as the MD, reduces available nutritional resources and transforms bacterial fermentation, favoring the growth of bacteria capable of utilizing carbohydrates. Therefore, dietary patterns can influence antibiotic resistance genes (ARGs) through bacterial interactions or extracellular metabolites [40]. In the future, dietary interventions aimed at controlling ARGs could become a tool for combating antibiotic resistance.

The effects of the MD on gut microbiota extend beyond increased functional diversity. Previous studies have shown variable results regarding individual bacterial genera [41]. In this study, statistically significant differences were observed in mean abundances at the genus level between HighMD and LowMD groups (Figure 6). In the HighMD group, increases were reported in genera such as *Paraclostridium*, *Anaerobutyricum*, *Romboutsia*, and *Butyricoccus*, anti-inflammatory butyrate-producing bacteria [42]. This finding is relevant, as butyrate plays a crucial role in gut health by providing energy to colon epithelial cells and modulating inflammation [43]. Notably, *Romboutsia* has been shown to decrease significantly during intestinal mucosal lesions, positioning it as a potential microbial biomarker for intestinal diseases [44].

In contrast, the LowMD group showed an increase in the genera *Lachnoanaerobacculum*, *Sporobacter*, and *Tepidibaculum*. Although the differences in genus abundances between HighMD and LowMD groups were significant, the specific implications of these changes remain unclear. The human gut microbiota is a dynamic and diverse ecosystem where bacteria interact in complex networks with functional redundancy. This complicates the assignment of exclusive roles to each taxon [45]. Therefore, further functional and experimental research is required to elucidate the implications of these observations.

At the family level, relevant differences were found. In the HighMD group, increases in relative abundances of *Enterobacteriaceae* and *Lachnospiraceae* were reported (Figure 4A). However, these differences were not statistically significant. Longitudinal studies in healthy individuals have shown that gut microbiota composition is relatively stable at high taxonomic levels. Nevertheless, significant strain-level turnover, particularly in *Enterobacteriaceae* populations, has been reported [46,47], which may explain the results obtained.

Regarding the WD, patterns observed in HighUPF and LowMD groups also revealed interesting differences. At the family level, relative abundances did not vary significantly between the two groups (Figure 4B). However, differences in β-diversity (Figure 5B) and α-diversity (Figure S4B) were identified.

Moreover, PCA under aerobic and anaerobic conditions (Figures S2 and S3) showed no correlation in metabolic diversity (Figure 1) or mean abundances at the genus level (Figure 7). These findings reinforce the idea that, while diet influences certain microbiota parameters, it interacts with other intrinsic and environmental factors contributing to the complexity of this ecosystem.

In summary, while the MD appears to be associated with beneficial changes in key butyrate-producing taxa, such as *Romboutsia* and *Butyricoccus*, the differences observed in the WD require more detailed analysis to understand its effects on gut microbiota and its relationship with host health.

### Strengths and Limitations

The observational design of the study does not allow conclusions on causality or directionality, as these results only indicate associations. Nevertheless, observational design is the most appropriate approach to explore such associations since longitudinal intervention-based studies, including high UPF intake and low MD adherence, could be harmful to health and pose ethical concerns. However, another limitation of the present study is the reduced sample size, along with the fact that due to the limited amount of sample, cenoantibiograms could not be performed in triplicates. A higher sample size would also increase the significance in bacterial comparisons, revealing differences in more bacterial genera.

Regarding the assessment of dietary intake, participant and social desirability biases are inherent limitations. Furthermore, the WD is complex and not solely characterized by the degree of food processing and UPF intake. The wide variety of food processing technologies and food additives available can exert varied effects on human health.

Finally, this study presents other technical limitations. The Biolog EcoPlate™ technique has not yet been extensively analyzed in human cecal samples, complicating its interpretation. This highlights the need to employ it in future studies for a better understanding of the functional metabolic capacity of gut microbiota. Moreover, the analysis of 16S rRNA using Nanopore also presents significant limitations, such as potential amplification biases and limited taxonomic resolution at the species level [48].

Nevertheless, this exploratory study presents numerous useful and promising insights, providing a valuable foundation for future research. Increasing the sample size and applying new methodologies would allow for a deeper understanding of the relationship between dietary patterns and the gut microbiota, helping to address the remaining questions in this field.

## 5. Conclusions

The results of this study suggest that adherence to the MD is associated with greater functional diversity in the gut microbiota of adolescents. However, no statistically significant differences in α-diversity were found between the two groups, indicating that microbial richness at the taxonomic level is not strongly influenced by adherence to the MD.

Despite the lack of differences in α-diversity, comparisons of microbial community structure between adolescents following the MD and those with high UPF intake showed clear differences in terms of β-diversity. These findings suggest that dietary patterns influence the composition of the gut microbiota in a more complex manner, beyond just taxonomic richness.

The observed differences in microbial response to antibiotics could be influenced by variations in microbial composition derived from dietary habits, highlighting the interaction between diet and gut microbiota in terms of resistance to antimicrobial treatments.

Regarding the abundance of specific bacterial genera, greater adherence to MD was associated with an increase in butyrate-producing genera, such as *Paraclostridium*, *Anaerobutyricum*, *Romboutsia*, and *Butyricoccus*, known for their anti-inflammatory effects and benefits for gut health. On the other hand, a significant decrease was observed in the genera *Sporobacter*, *Lachnoanaerobaculum*, and *Tepibaculum*, which could reflect a more favorable microbial pattern for the prevention of inflammatory diseases.

These results support the idea that adherence to the MD promotes a balanced gut microbiota, favoring the production of beneficial metabolites such as butyrate.

## Figures and Tables

**Table 1:** General characteristics of the study population.

		Total		Male		Female	
		*n*	%	*n*	%	*n*	%
Total Population		19	100	9	47.4	10	52.6
Adherence to MD (KIDMED test [16])	Low MD	8	42.1	4	21.1	4	21.1
	High MD	11	57.9	5	26.3	6	31.6
UPF intake (SQ-HPF test [17])	Low UPF	9	47.4	6	31.6	3	15.8
	High UPF	10	52.6	3	15.8	7	36.8

MD: Mediterranean diet; UPF: ultra-processed foods.

## Data Availability

The original contributions presented in this study are included in the article. Further inquiries can be directed to the corresponding author.

## Supplementary Materials

The supporting information can be downloaded at https://www.mdpi.com/article/10.3390/nu17030388/s1.

## Abbreviations

The following abbreviations are used in this manuscript:

AK	Amikacin
AML	Amoxicillin
ARG	Antibiotic resistance genes
AUG	Amoxicillin/clavulanic acid
AWCD	Average well color development
AZM	Azithromycin
BC	Bray–Curtis
CAZ	Ceftazidime
CD	Clindamycin
CIP	Ciprofloxacin
CN	Gentamicin
CR	Cefpirome
FOX	Cefoxitin
IMI	Imipenem
IMI+EDTA	Imipenem + EDTA
LEV	Levofloxacin
MD	Mediterranean diet
MIC	Minimum inhibitory concentration
MTZ	Metronidazole
PCA	Principal component analysis
PCoA	Principal coordinates analysis
RD	Rifampicin
SCFAs	Short-chain fatty acids
TS	Sulfamethoxazole/trimethoprim
UPF	Ultra-processed foods
WD	Western diet
