# Supplementary material for: Cross-Sectional Comparative Analysis of Gut Microbiota in Spanish Adolescents with Mediterranean and Western Diets
Source: Nutrients. 2025 Jan 22;17(3):388. doi: 10.3390/nu17030388 (PMC11820480; doi:10.3390/nu17030388)
Supplement: Supplementary file 1 [file nutrients-17-00388-s001.zip › nutrients-3417474-supplementary.pdf]

Cross-sectional comparative analysis of gut microbiota in Spanish adolescents with Mediterranean and Western diets

Supplementary material

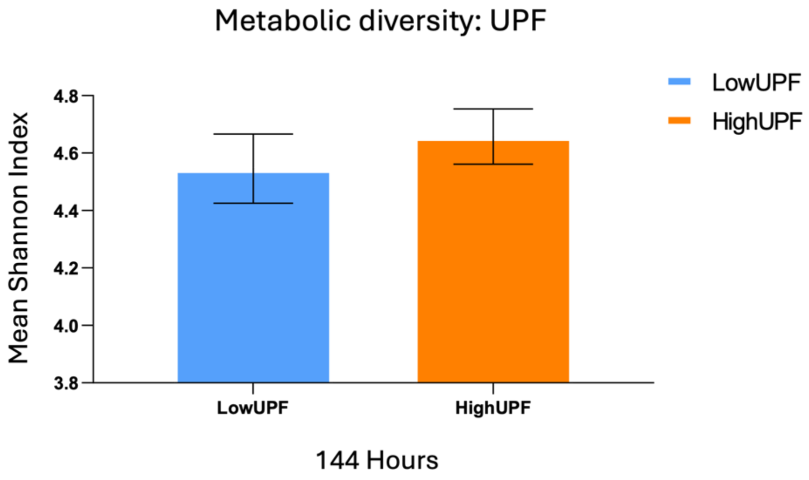

Figure S1. Metabolic diversity of gut microbiota according to the degree of UPF intake. Bar chart for the two UPF intake groups represented on the X-axis, measured after 144 hours. The Y-axis shows the mean values of the Shannon-Weaver index, with error bars indicating variability within each group. UPF: ultra-processed foods.

Principal Component Analysis in Aerobic Conditions: UPF

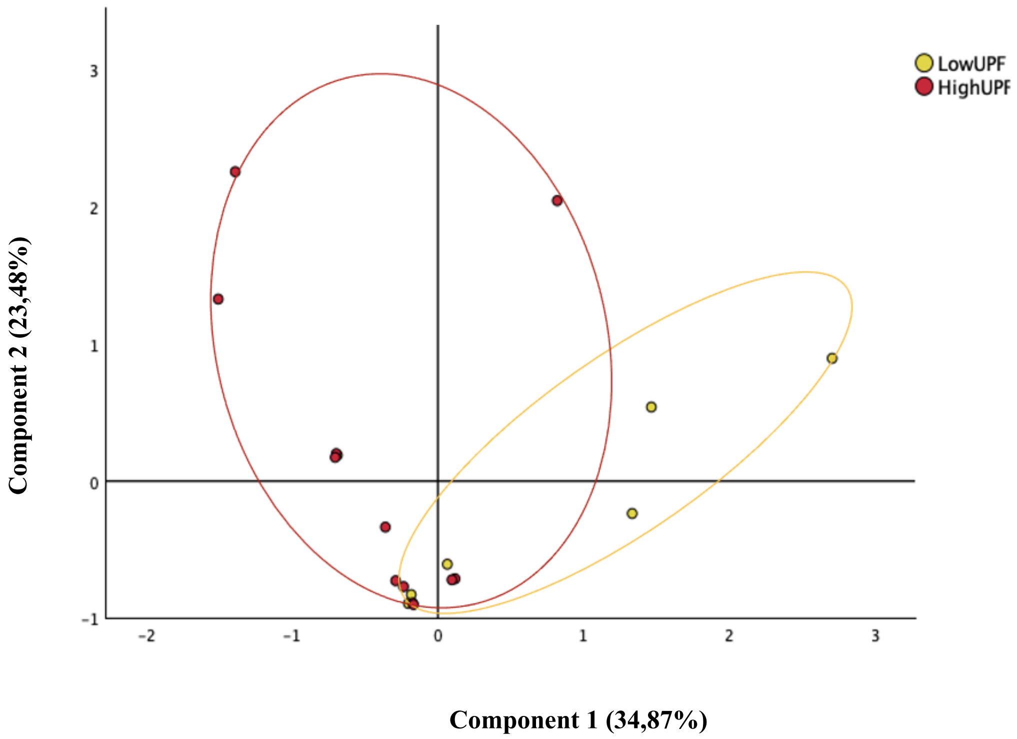

Figure S2. Antibiotic response under aerobic conditions according to the degree of UPF intake. The PCA plot shows Component 2 on the Y-axis, accounting for 23.48% of the total variability in the data. The X-axis (Component 1) explains 34.87% of the data variability. Each point represents an individual observation, categorized into two groups: HighUPF and LowUPF. The ellipses on the plot represent confidence regions that contains approximately 95% observations for each group. PCA: Principal component analysis; UPF: ultra-processed foods.

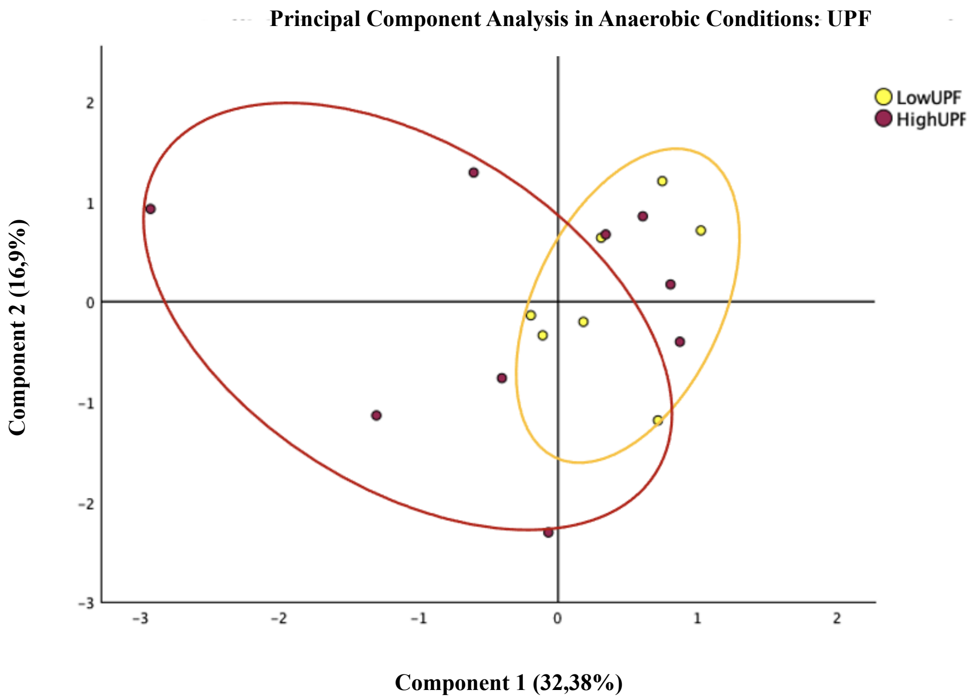

Figure S3. Antibiotic response under anaerobic conditions according to the degree of UPF intake. The PCA plot under anaerobic conditions shows Component 2 on the Y-axis, accounting for 16.9% of the total data variability. The X-axis (Component 1) accounts for 32.28% of the data variability. Each point represents an individual observation, categorized into two groups: HighUPF and LowUPF. The ellipses on the graph indicate the confidence regions that contain approximately 95% of the observations for each group. PCA: Principal component analysis; UPF: ultra-processed foods.

Table S1. Mean MICs (µg/mL) under aerobic conditions for all participants, classified into MD and UPF groups.

SQHPF	KIDMED	AML	AK	CAZ	CN	TS	CR
High UPF	Low MD	256	1	0.094	0.25	0.32	0.032
Low UPF	High MD	1.5	24	0.094	4	0.064	0.064
Low UPF	High MD	0.75	8	256	2	0.5	6
Low UPF	Low MD	1.5	8	24	0.5	0.064	16
High UPF	Low MD	256	1	0.19	0.19	0.047	0.25
High UPF	High MD	48	0.38	0.094	0.064	0.032	0.047
Low UPF	Low MD	256	0.75	0.125	0.25	0.094	0.75
High UPF	Low MD	4	1	0.047	0.125	0.032	0.047
High UPF	High MD	256	1	0.125	0.25	16	0.25
High UPF	High MD	16	1.5	0.125	1	0.012	0.064
High UPF	High MD	256	12	256	2	0.032	64
Low UPF	High MD	6	1.5	0.094	0.19	0.75	0.125
High UPF	Low MD	32	0.5	0.094	0.125	0.016	2
Low UPF	High MD	0.75	256	192	2	0.047	2
High UPF	High MD	128	0.25	0.125	0.38	0.094	0.094
High UPF	Low MD	8	12	0.047	0.75	0.094	4
Low UPF	High MD	8	0.38	0.094	0.064	0.064	0.025
High UPF	High MD	6	1	0.064	0.125	0.064	0.047
High UPF	Low MD	256	0.38	0.5	0.047	1.5	256

MICs: Minimum inhibitory concentrations; MD: Mediterranean diet; UPF: ultra-processed foods.

Table S2. Mean MICs (μg/mL) under anaerobic conditions for all participants, classified into MD and UPF groups.

SQ-HPF	KIDMED	AUG	LEV	FOX	CD	RD	MTZ	AZM	CIP	IMI + EDTA	IMI
Low UPF	Low MD	16	2	8	1	1	32	16	0.25	0.094	>0.125
Low UPF	High MD	32	2	32	1	4	32	16	1	0	0
High UPF	High MD	16	0.5	32	1	1	32	32	0.5	0.25	>0.125
High UPF	Low MD	8	0.5	32	1	1	16	32	1	0.25	3
Low UPF	High MD	8	2	32	0.5	4	32	32	0.5	0	0
High UPF	Low MD	32	1	32	1	4	32	32	1	0.064	>0.125
High UPF	High MD	32	2	32	1	4	16	16	1	0	0
High UPF	High MD	8	0.5	8	1	4	32	32	1	>0.032	>0.125
Low UPF	High MD	32	0.5	32	1	1	32	32	0.5	0	1.5
Low UPF	High MD	32	2	32	1	4	32	16	0.25	>0.032	>0.125
High UPF	High MD	8	0.5	32	1	1	16	16	0.25	2	8
Low UPF	High MD	32	2	32	0.25	1	32	32	1	0.38	1.5
Low UPF	Low MD	32	1	8	1	2	32	32	1	>0.032	>0.125
High UPF	Low MD	16	2	32	0.25	1	32	16	0.5	1	0
High UPF	Low MD	32	0.5	32	1	2	32	16	0.25	>0.032	>0.125
High UPF	Low MD	32	0.5	32	1	4	32	16	1	0.047	>0.125

MICs: Minimum inhibitory concentrations; MD: Mediterranean diet; UPF: ultra-processed foods.

Table S3. Relative abundances for MD groups.

Phylum	***LowMD***	***HighMD***
Firmicutes	84%	86,5%
Bacteroidetes	8,4%	7,95%
Proteobacteria	6,9%	5,25%
Verrucomicrobiota	0,5%	0,51%
Family	***LowMD***	***HighMD***
Oscillospiraceae	34,12%	30,32%
Lachnospiraceae	26,44%	30,87%
Veillonellaceae	9,98%	4,69%
Bacateridaceae	6,96%	9,77%
Peptostreptococcacea	2,72%	5,59%
Enterobacteriaceae	2,8%	4%
Akkermansiaceae	3%	0,38%
Order	***LowMD***	***HighMD***
Eubacterial	77,55%	78,7%
Bacteroidales	7,82%	9,71%
Enterobacterials	2,23%	3,12%
Veillonellales	7,69%	3,71%
Lactobacillales	0,6%	1,22%
Verrucomicrobials	2,7%	0,27%
Gender	***LowMD***	***HighMD***
Other	55,48%	60%
Eubacterium	5,63%	9,27%
Gemmiger	8,21%	7,97%
Blautia	6,15%	6,25%
Dialister	7,21%	2,77%
Ruminococcus	4,35%	3,13%
Vescimonas	3,78%	2,99%

MD: Mediterranean diet.

Table S4. Relative abundances for UPF groups.

Phylum	*LowUPF*	*HighUPF*
Firmicutes	86,38%	84,93%
Bacteroidetes	7,83%	7,95%
Proteobacteria	5,14%	7,95%
Verrucomicrobiota	0,46	0,53%
Family	***LowUPF***	***HighUPF***
Oscillospiraceae	36,45%	29,28%
Lachnospiraceae	25,18%	31,24%
Veillonellaceae	1,65%	10%
Bacateridaceae	7,8%	9%
Peptostreptococcacea	5,73%	3,59%
Enterobacteriaceae	7,86%	0,92%
Akkermansiaceae	3,38%	0,42%
Order	***LowUPF***	***HighUPF***
Eubacterial	77,45%	78,66%
Bacteroidales	8,35%	9,24%
Enterobacterials	6,21%	0,7%
Veillonellales	1,41%	7,70%
Lactobacillales	1,12%	0,87%
Verrucomicrobials	3,02%	0,29%
Gender	***LowUPF***	***HighUPF***
Other	65,5%	54,4%
Eubacterium	8,08%	7,54%
Gemmiger	7,39%	8,47%
Blautia	5,47%	6,63%
Dialister	1,11%	6,71%
Ruminococcus	2,11%	4,53%
Vescimonas	2,94%	3,55%

UPF: ultra-processed foods.

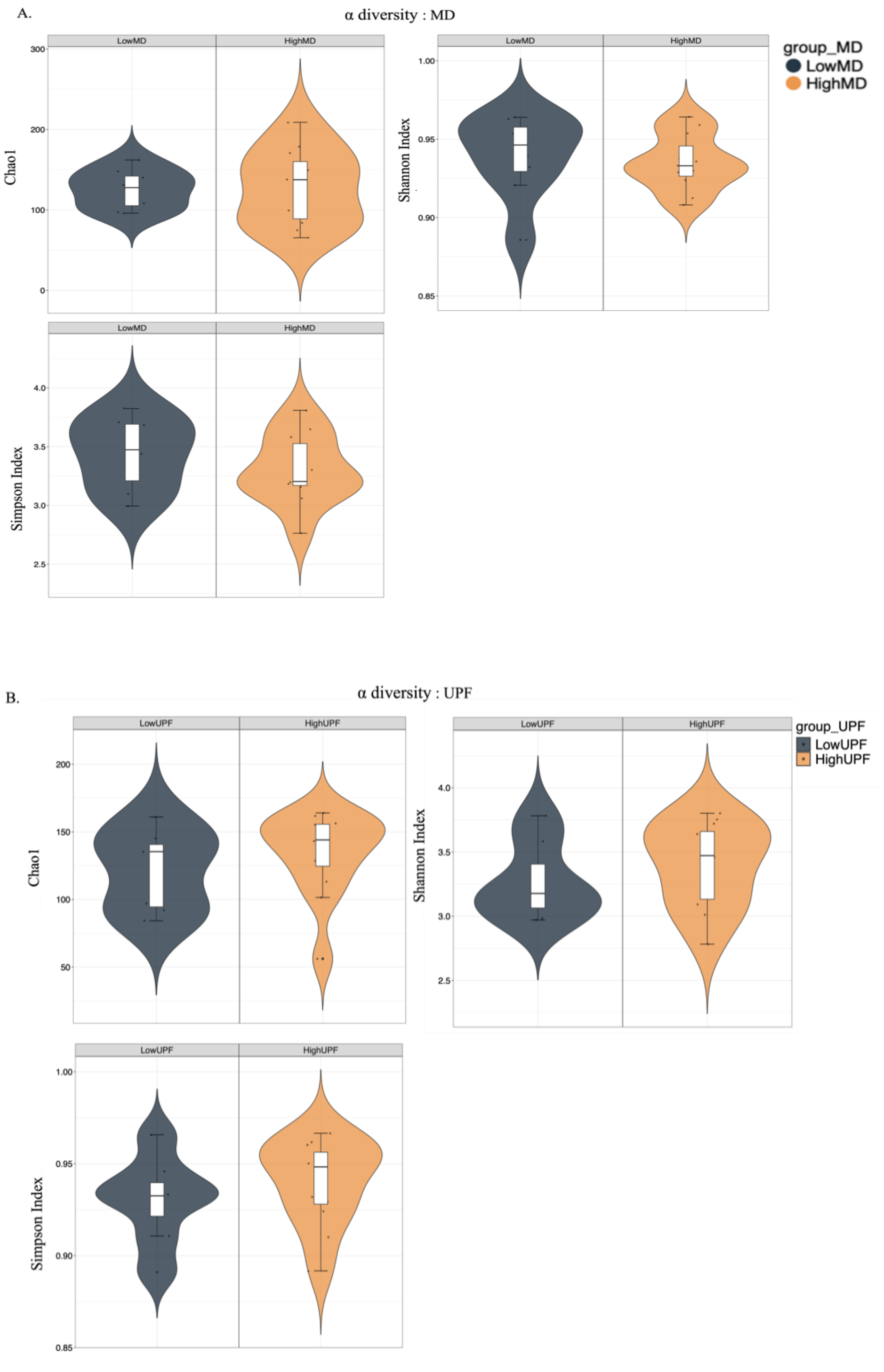

Figure S4. α-diversity for (A) MD and (B) UPF groups. The Y-axis shows the type of index (Chao1, Shannon and Simpson), representing the richness, diversity and equity and dominance of species, respectively. While the X-axes categorize the samples according to food intake (HighUPF, LowUPF, HighMD and LowMD). Values outside this range are considered outliers and are represented as individual points. MD: Mediterranean diet; UPF: ultra-processed foods.

Table S5. STROBE Statement

	Item No	Recommendation	Page No
**Title and abstract**	1	(*a*) Indicate the study's design with a commonly used term in the title or the abstract	1
		(*b*) Provide in the abstract an informative and balanced summary of what was done and what was found	1
**Introduction**			
Background/rationale	2	Explain the scientific background and rationale for the investigation being reported	2
Objectives	3	State specific objectives, including any prespecified hypotheses	2
**Methods**			
Study design	4	Present key elements of study design early in the paper	3
Setting	5	Describe the setting, locations, and relevant dates, including periods of recruitment, exposure, follow-up, and data collection	3
Participants	6	(*a*) Give the eligibility criteria, and the sources and methods of selection of participants	3
Variables	7	Clearly define all outcomes, exposures, predictors, potential confounders, and effect modifiers. Give diagnostic criteria, if applicable	3-4
Data sources/ measurement	8*	For each variable of interest, give sources of data and details of methods of assessment (measurement). Describe comparability of assessment methods if there is more than one group	*3-4*
Bias	9	Describe any efforts to address potential sources of bias	3-4
Study size	10	Explain how the study size was arrived at	3
Quantitative variables	11	Explain how quantitative variables were handled in the analyses. If applicable, describe which groupings were chosen and why	3-4
Statistical methods	12	(*a*) Describe all statistical methods, including those used to control for confounding	4
		(*b*) Describe any methods used to examine subgroups and interactions	-
		(*c*) Explain how missing data were addressed	-
		(*d*) If applicable, describe analytical methods taking account of sampling strategy	-
		(*e*) Describe any sensitivity analyses	-
**Results**			
Participants	13*	(a) Report numbers of individuals at each stage of study—eg numbers potentially eligible, examined for eligibility, confirmed eligible, included in the study, completing follow-up, and analysed	-
		(b) Give reasons for non-participation at each stage	-
		(c) Consider use of a flow diagram	-
Descriptive data	14*	(a) Give characteristics of study participants (eg demographic, clinical, social) and information on exposures and potential confounders	5

		(b) Indicate number of participants with missing data for each variable of interest	3
Outcome data	15*	Report numbers of outcome events or summary measures	-
Main results	16	(*a*) Give unadjusted estimates and, if applicable, confounder-adjusted estimates and their precision (eg, 95% confidence interval). Make clear which confounders were adjusted for and why they were included	-
		(*b*) Report category boundaries when continuous variables were categorized	3
		(*c*) If relevant, consider translating estimates of relative risk into absolute risk for a meaningful time period	-
Other analyses	17	Report other analyses done—eg analyses of subgroups and interactions, and sensitivity analyses	-

Discussion

Key results	18	Summarise key results with reference to study objectives	13-16
Limitations	19	Discuss limitations of the study, taking into account sources of potential bias or imprecision. Discuss both direction and magnitude of any potential bias	15-16
Interpretation	20	Give a cautious overall interpretation of results considering objectives, limitations, multiplicity of analyses, results from similar studies, and other relevant evidence	13-16
Generalisability	21	Discuss the generalisability (external validity) of the study results	15-16

Other information

Funding	22	Give the source of funding and the role of the funders for the present study and, if applicable, for the original study on which the present article is based	17
